# Egg discrimination along a gradient of natural variation in eggshell coloration

**DOI:** 10.1098/rspb.2016.2592

**Published:** 2017-02-08

**Authors:** Daniel Hanley, Tomáš Grim, Branislav Igic, Peter Samaš, Analía V. López, Matthew D. Shawkey, Mark E. Hauber

**Affiliations:** 1 Department of Biology, Long Island University - Post, Brookville, NY 11548-1300, USA; 2 Department of Zoology and Laboratory of Ornithology, Palacký University, Olomouc 77146, Czech Republic; 3 Department of Biology, University of Akron, Akron, OH 44325, USA; 4 Division of Ecology and Evolution, Research School of Biology, Australian National University, Canberra 2601, Australia; 5 Departamento de Ecología, Genética y Evolución, Facultad de Ciencias Exactas y Naturales, Universidad de Buenos Aires, C1428EHA Buenos Aires, Argentina; 6 Department of Biology, Evolution and Optics of Nanostructures Group, Ghent University, Ghent 9000, Belgium; 7 Department of Psychology, Hunter College and the Graduate Center of the City University of New York, New York, NY 10065, USA; 8 Department of Animal Biology, School of Integrative Biology, University of Illinois at Urbana-Champaign, Urbana, IL 61801, USA

**Keywords:** brood parasitism, colour perception, decision-making, egg discrimination, recognition

## Abstract

Accurate recognition of salient cues is critical for adaptive responses, but the underlying sensory and cognitive processes are often poorly understood. For example, hosts of avian brood parasites have long been assumed to reject foreign eggs from their nests based on the total degree of dissimilarity in colour to their own eggs, regardless of the foreign eggs' colours. We tested hosts' responses to gradients of natural (blue-green to brown) and artificial (green to purple) egg colours, and demonstrate that hosts base rejection decisions on both the direction and degree of colour dissimilarity along the natural, but not artificial, gradient of egg colours. Hosts rejected brown eggs and accepted blue-green eggs along the natural egg colour gradient, irrespective of the total perceived dissimilarity from their own egg's colour. By contrast, their responses did not vary along the artificial colour gradient. Our results demonstrate that egg recognition is specifically tuned to the natural gradient of avian eggshell colour and suggest a novel decision rule. These results highlight the importance of considering sensory reception and decision rules when studying perception, and illustrate that our understanding of recognition processes benefits from examining natural variation in phenotypes.

## 1. Introduction

The recognition of suitable food, mates, predators, and shelter is central to all life. An organism's fitness depends on its ability to recognize phenotypic differences that can vary from obvious to nearly imperceptible [1,2]. However, decision-making in a natural context can be challenging because novel stimuli inevitably differ from previously encountered stimuli. Here, we used avian brood parasite–host interactions as a tractable system to explore the perceptual bases of these recognition processes in the wild.

Avian brood parasites lay their eggs into other birds' nests and impose the cost of rearing their young upon host parents [3–5]. Hosts evade these costs by preventing parasitism [6] or rejecting parasitic eggs or young from their nests [3,4]. As hosts evolve better discrimination abilities, selection favours parasites with eggs that more accurately mimic host egg appearance [7,8], which can lead to coevolutionary arms races [9]. Prior experience with brood parasitism affects an individual's response [10–12]; experience with their own and foreign eggs provides hosts with valuable information on a range of egg phenotypes that will allow for more flexible future decisions (e.g. [12]). One common host defence is to reject a parasitic egg that differs from a learned or innate internal template of the host's own eggshell appearance [10,13] and eggshell coloration and maculation (i.e. spotting) are the primary cues that most hosts use for such egg recognition tasks [4].

**Figure 1.**
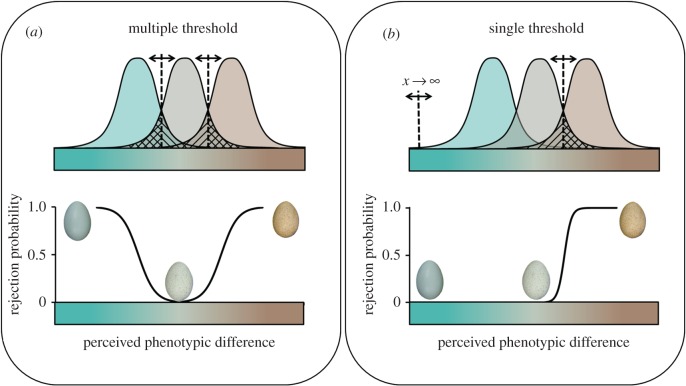
Decision-making by hosts of avian brood parasites is an ideal system for studying general principles of cognition in nature. These hosts must detect and appropriately respond to a brood parasite's trickery while balancing the risk of acceptance or rejection errors (striped and cross-hatched areas, respectively). The acceptance threshold (dashed vertical lines) lies at the intersection of these risks [14], such that stimuli between both thresholds are accepted and beyond which stimuli are rejected. These thresholds can shift (infinitely far) depending on perceived risk (bi-directional arrows on acceptance thresholds), making them akin to decision boundaries in general recognition theory [15] rather than demarcating a host's perceptual limits (i.e. psychological versus psychophysical). In the top portion of each schematic (*a,b*) we illustrate a distribution of host eggshell phenotypes (middle) and distributions for two parasites (left and right). The traditional expectation based on multiple thresholds (*a*, bottom) is that as the magnitude of perceived difference between host and parasitic eggs increases hosts are more likely to respond; therefore, blue-green and brown parasitic eggs that are equally different to the host's eggs should be rejected at equal rates. However, if hosts base rejection decisions on (*b*) specific colours, then we expect (*b*, bottom) that rejections would be biased toward one end of the phenotypic range, despite the absolute perceived difference; for example, such that either blue-green or brown parasitic eggs are rejected.

Most studies (electronic supplementary material, table S1) have examined host responses based on the *absolute* perceived colour dissimilarity between host and parasitic eggs (hereafter, the multiple threshold decision rule; figure 1*a*). However, hosts may be biased toward rejecting eggs with colours at either end of their phenotypic range (hereafter, the single threshold decision rule, figure 1*b*), rather than having their responses governed only by the magnitude of the perceived difference (at both tails of a host's phenotypic range; figure 1*a*). Birds' eggshell colours are ideally suited for testing if host responses are governed by single or multiple discrimination thresholds because they vary linearly from blue-green to brown through the avian colour space [16]. Some studies that have examined the role of each of birds' four individual photoreceptors found that variation in perceived ultraviolet and blue light predicted host egg rejection behaviour while absolute perceived colour differences did not [17]. This suggests that perceived variation in specific colours may have governed their rejection responses, which might be adaptive if hosts have either a learned or innate aversion to parasitic egg colours. Thus, despite vast research [4], the decision rules underlying colour-based parasitic egg recognition remain unclear.

**Figure 2.**
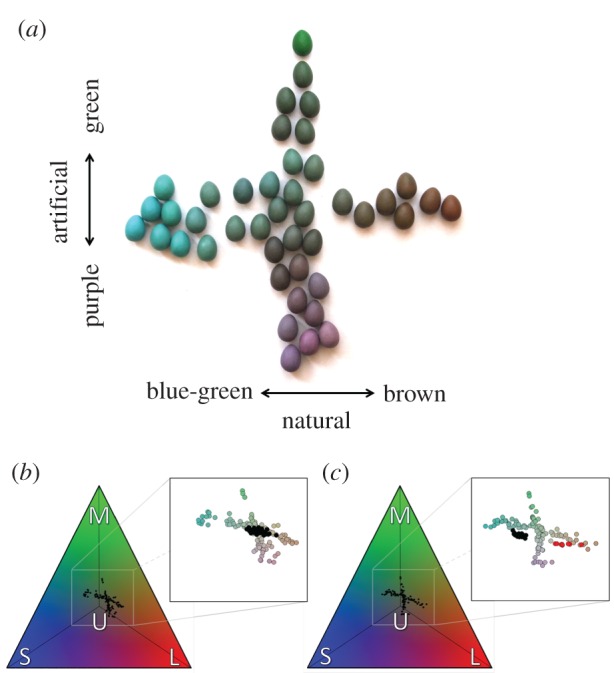
Foreign eggs were (*a*) painted across two gradients of variation that either align (blue-green to brown) or are orthogonal (green to purple) with natural eggshell colours. These manipulations produced perceivable colour variation that represents relevant threats and novel stimuli to hosts (see the electronic supplementary material). These models, presented to (*b*) blackbirds and (*c*) robins, were specifically designed with respect to the avian tetrahedral colour space (shown from above). Within each tetrahedron we illustrate the predicted short ‘S’, medium ‘M’, long ‘L’, and ultraviolet ‘U’ wavelength-sensitive photoreceptor stimulation when these foreign eggs are viewed by the host. Insets show these models (in actual colour) alongside variation of natural (*b*) blackbird and (*c*) robin eggshell colours (black dots within each inset). For the purpose of comparison, we show eggshell coloration of the brown-headed cowbird (red dots in *c*) that parasitizes the robin (data from [16]).

To experimentally test whether hosts employ a single threshold decision rule, we painted foreign eggs to vary continually along two colour gradients within the avian perceptual colour space representing either natural or artificial egg colours (figure 2). The first gradient encompassed natural variation in birds' eggshell colours, which varies from blue-green to brown [16]. The second gradient encompassed a range of artificial colours, orthogonal to the first within the host's visual space, varying from green to purple (figure 2). These foreign eggs were added to the nests of blackbirds *Turdus merula* (hereafter, blackbird) and American robins *T. migratorius* (hereafter, robin) and we recorded whether these hosts accepted or rejected the foreign eggs from their nests. If host rejection decisions are based solely on absolute perceived colour differences, their responses should be independent of the direction of the colour differences (figure 1*a*) and similar along both colour gradients. By contrast, if hosts use a single threshold decision rule (figure 1*b*), then we expect predictable responses only along the natural egg colour gradient because these represent relevant stimuli [16]. Finally, we more fully explored these hosts' responses by quantifying and comparing host discrimination abilities.

## 2. Material and methods

### (a) Study area and experimental procedures

We studied blackbirds in Olomouc, Czech Republic (49°36′ N, 17°15′ E) and robins in Ithaca, New York, USA (42°26′ N, 76°30′ W) between April and July 2014, and successfully finished experiments at 82 blackbird and 52 robin nests. Conspecific parasitism rates for blackbirds in our population are conservatively estimated at 3.1% [18] and cuckoos do not parasitize this population because cuckoos avoid towns [19]. Our robin population is sympatric with cowbirds [20,21] and may experience cowbird parasitism, as indicated by the presence of a cowbird egg in an abandoned robin nest [22]. We introduced a single foreign egg model into each nest and recorded whether or not the attending female was flushed from the nest [23]; these eggs were unspotted immaculate (i.e. unspotted) and their colours uniquely positioned along a gradient of blue-green to brown colour variation representative of natural avian eggshell colours [16] or an alternative orthogonal gradient varying from green to purple (figure 2; electronic supplementary material, figure S1). After each egg introduction, we monitored the nest daily for six consecutive days [24]. Hosts were considered ‘rejecters' when the foreign egg or one of their own eggs disappeared from their nests during this six-day period. To ensure rejection responses were possible, these eggs were consistent in size, shape, and material with experimental eggs previously used in these populations (see the electronic supplementary material), differing only in their colour. We did not detect conspecific or interspecific parasitism in any of these nests (for further details, see the electronic supplementary material).

### (b) Colour analysis

We used reflectance spectrometry to objectively measure the coloration of freshly abandoned eggs from both hosts, and also foreign egg models. Then, using visual information of the blackbird [25] and a noise-limited visual model [26], we calculated the perceived chromatic and achromatic contrast in units of just noticeable difference (hereafter JND) between the average host colour and each egg model. Under ideal viewing conditions a JND < 1 represents an imperceptibly small difference between the hosts' eggs and the foreign egg, while a JND of one would be just noticeable under ideal viewing conditions, and JNDs > 1 become increasingly noticeable as the JNDs increase. We then summarized perceivable variation in colour using perceptually uniform chromaticity diagrams [27], which allowed for examining both the direction and degree of JNDs. For further details, see the electronic supplementary material.

### (c) Statistical analyses

We used binomial generalized linear models (GLM) to predict each host's response (accept or reject), using the ‘*glm*’ function in the base ‘*stats*' package in R v. 3.1.2 [28]. We decided to use a logit link function to ensure our results are comparable with previous studies that have widely used this link function to describe host responses (e.g. [13,23,29]); however, other parametric psychometric functions (e.g. Gaussian or Weibull) could also explain host responses. Therefore, to ensure our results were robust to the form of psychometric function, we reran the GLM using the appropriate link function for each psychometric alternative (the probit link function for Gaussian and the complementary log–log function for Weibull) [30]. We report the threshold location as the colour value associated with a rejection probability of 0.50, based on models refitted with only the predictor of interest [30,31]. This describes the location of each host's decision boundary, along either colour gradient (in JND units in a particular direction) or across differences in absolute dissimilarity (in JND units), which we report as the median and inter-quartile range based on 10 000 bootstrap estimates. We also present Nagelkerke's *R*^2^ and the small sample size-corrected Akaike's Information Criterion AICc [32,33].

First, we examined if both chromatic and achromatic contrast predicted host response (multiple threshold decision rule). Then, we predicted host response by the three gradients of manipulated colour variation, controlling for the perceived achromatic contrast (single threshold decision rule). For these models, we report the evidence ratio [34], in which unlike AIC_c_ weights do not depend on the alternative models and which quantifies the empirical support for one hypothesis over an alternative hypothesis [34,35].

In addition, we used an information-theoretic (I-T) approach [34] to produce an average model that would incorporate the uncertainty of many similarly probable alternatives and identify the models that best described the variation in our data [36]. Specifically, we produced a global GLM predicting host response by our main variables of interest, which were the three gradients of colour variation, chromatic and achromatic contrast, as well as other variables with the potential to impact host response [18,23,37]: whether they were flushed from their nest (categorical: yes or no), final clutch size (continuous), laying date (continuous), and nest age (continuous). We then established a candidate set based on the relative likelihood of potential models such that models with evidence ratios greater than 1/8 were considered reasonable [34]. We averaged models in this candidate set using the ‘*MuMIn*’ package v. 1.13.4 [38]. The relative importance of each predictor of host response was calculated as the sum of AIC_c_ weights over all the models in the candidate set where that predictor occurs, setting the effect of a parameter at zero if it was not included in a particular model within the candidate set, to avoid biasing our model averaged estimates away from zero [34].

To examine if blackbirds and robins expressed different discrimination abilities to experimental parasitism, we compared the slopes of their predicted responses along the natural eggshell colour gradient (i.e. regression coefficients for responses to variation along the blue-green to brown gradient). Using a resampling approach [39], we randomly selected 90% of the blackbird and robin data, respectively, and reran GLMs (see above) separately for each species using these data, recording the regression coefficients (i.e. slopes) for blue-green to brown variation 10 000 times. Normality of the resampled populations was tested using Kolmogorov–Smirnov tests, using 1 000 Monte Carlo simulations [40], and neither population was normally distributed (blackbird: Kolmogorov–Smirnov test = 0.61, bootstrap *p* < 0.0001, Monte Carlo simulations = 1 000; robin: Kolmogorov–Smirnov test = 0.97, bootstrap *p* < 0.0001, Monte Carlo simulations = 1 000). Therefore, we tested for differences in slopes using a Wilcoxon rank sum test and report the rank-biserial correlation [41].

All analyses were conducted in R v. 3.1.2 [28]. For more complete details on the methods and statistical analyses used, see the electronic supplementary material.

## 3. Results

**Figure 3.**
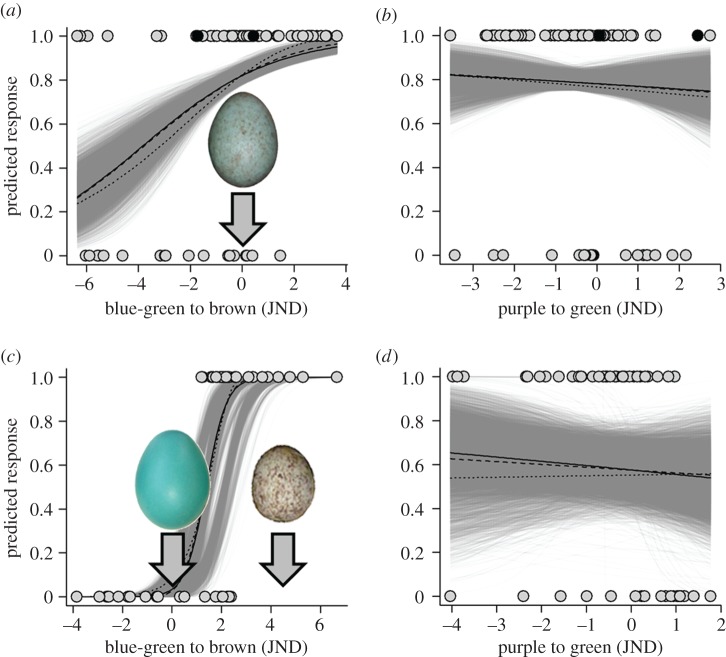
The probability of rejecting a coloured foreign egg is shown for (*a,b*) blackbirds (*n* = 82) and (*c*,*d*) robins (*n* = 52), with respect to the position of each hosts' own egg colour (see inset eggs above zero on both *x*-axes) along the (*a,c*) blue-green to brown and (*b,d*) purple to green colour gradients (in JNDs). We show a significant logistic (solid line, table 1), Gaussian (dashed, electronic supplementary material, table S2), and Weibull (dotted, electronic supplementary material, table S3) fits. Please note, we plotted all egg rejections, including rejection errors (black dots; *n* = 2) and foreign eggs falling along both colour dimensions. For comparison, we plotted (*c*) the mean location (approx. 4 JND on the *x*-axis) of eggshell coloration along this axis for the robin's heterospecific brood parasite, the brown-headed cowbird (also see figure 2). We illustrate 10 000 resampled slopes from binomial models predicting host behavioural responses (light grey lines); refer to table 1 for the significance of these parameters.

We found that both hosts' rejection responses varied predictably across the gradient of natural eggshell colours. This natural eggshell colour gradient had the greatest relative importance of any potential predictive variable (table 2); no other parameter could effectively predict either host's response. Blackbirds rejected eggs browner than their own at higher rates (mean ± standard error (s.e.): 86.96 ± 0.61%) than eggs that were more blue-green than their own (66.00 ± 3.18%, threshold location: median = −3.84 JND more blue-green; inter-quartile range = 1.56 JND; figure 3 and table 1). Robins also rejected eggs browner than their own (78.45 ± 3.26%) at higher rates than eggs more blue-green than their own (0.40 ± 0.22%; threshold location: median = 1.16 JND browner, inter-quartile range = 0.33 JND; figure 3 and table 1). By contrast, neither the blackbird's (threshold location: median = 2.41 JND greener, inter-quartile range = 2.60 JND) nor the robin's responses were predicted across the gradient of artificial eggshell colours (threshold location: median = 0.51 JND greener, inter-quartile range = 0.63 JND; figure 3 and table 1). Moreover, blackbird rejection responses were negatively (not positively) related to the absolute perceived degree of dissimilarity, i.e. chromatic contrast (threshold location: median = 5.49 JND, inter-quartile range = 1.82 JND; figure 3*a* and table 1), while robin responses were unrelated (threshold location: median = 2.30 JND, inter-quartile range = 0.55 JND; figure 3*b* and table 1). Alternative psychometric functions produced equivalent results (figure 3; also see electronic supplementary material, tables S2 and S3). These findings provide strong support that these hosts use a single threshold decision (figure 1*b*) rather than the traditionally assumed multiple threshold decision rule (figure 1*a*, figure 3, and table 1).

**Table 1.** Generalized linear models predicting the rejection probability of foreign eggs by blackbirds and robins. For each species, we tested the predictions of the multiple threshold and single threshold decision rule scenarios. Whole model statistics including Nagelkerke's *R*^2^, *AICc*, and *AICc* weight (*w_i_*) are presented. We show parameter estimates, their standard errors (s.e.), 95% lower and upper confidence limits (LCL and UCL), a measure of standardized effect (*z*-score), and their variance inflation factors (VIF). All parameter estimates represent the change in log-odds of egg rejection for an increase of one just noticeable difference (JND). Significant models and effects are italicized. *χ*^2^ is chi-squared.

host	scenario	parameter	estimate	s.e.	LCL	UCL	*z*	*χ*^2^	d.f.	*p*	VIF
Blackbird^a^	*multiple threshold (χ^2^ = 6.90, R^2^ = 0.12, AICc = 90.54, w_i_ = 0.20, n = 82, p = 0.03*)										
		(intercept)	0.88	1.52	−2.14	3.91	0.58	—	1	0.56	—
		*chromatic contrast*	*−0.36*	*0.18*	*−0.73*	*<−0.001*	*−1.94*	*3.84*	*1*	*0.05*	*1.10*
	* *	achromatic contrast	0.06	0.06	−0.06	0.19	1.00	1.04	1	0.31	1.10
	*single threshold (χ^2^ = 14.14, R^2^ = 0.24, AICc = 87.76, w_i_ = 0.80, n = 82, p < 0.01*)										
		(intercept)	0.97	1.50	−1.96	4.00	0.65	—	1	0.52	—
		*blue-green to brown*	*0.40*	*0.16*	*0.11*	*0.73*	*2.53*	*7.44*	*1*	*<0.01*	*1.83*
		green to purple	−0.07	0.27	−0.61	0.48	−0.25	0.06	1	0.80	1.60
		less UV to more UV	−0.28	0.55	−1.39	0.79	−0.52	0.27	1	0.60	1.56
* *	* *	achromatic contrast	0.01	0.07	−0.12	0.15	0.21	0.04	1	0.83	1.38
Robin^a^	*multiple threshold* (*χ*^2^ = 5.92, *R*^2^ = 0.15, *AICc* = 68.86, *w_i_* < 0.0001, *n* = 52, *p* = 0.05)										
		(intercept)	−1.86	1.90	−5.81	1.81	−0.98	—	1	0.33	—
		*chromatic contrast*	*0.83*	*0.39*	*0.14*	*1.71*	*2.12*	*5.86*	*1*	*0.02*	*1.00*
	* *	achromatic contrast	<−0.01	0.09	−0.19	0.18	−0.02	<0.01	1	0.98	1.00
	*single threshold (χ^2^ = 35.29, R^2^ = 0.67, AICc = 44.29, w_i_ = 1.00, n = 52, p < 0.0001*)										
		(intercept)	0.24	3.36	−7.11	6.89	0.07	—	1	0.94	—
		*blue-green to brown*	*2.43*	*1.02*	*1.00*	*5.19*	*2.37*	*27.37*	*1*	*<0.0001*	*1.59*
		green to purple	−0.08	0.36	−0.78	0.67	−0.23	0.05	1	0.82	1.39
		less UV to more UV	−1.74	1.06	−4.17	0.07	−1.64	3.50	1	0.06	1.90
* *	* *	achromatic contrast	−0.36	0.23	−0.89	0.03	−1.60	3.26	1	0.07	1.46

^a^Evidence ratios show that the single threshold decision rule explains blackbird response four times better and robin responses 200 000+ times better than the multiple threshold decision rule.

**Table 2.** Averaged parameter estimates from generalized linear models (see table 1) with their adjusted standard errors (s.e.) [34], 95% lower and upper confidence intervals (LCL and UCL), measures of standardized effect (*z*-score), and relative importance. Estimates are presented as changes in log-odds of rejecting an egg for an increase of one JND. Host response to foreign egg model (either accept or reject) was predicted by the axes of colour variation (blue-green to brown, green to purple, less UV to more UV), chromatic and achromatic contrast (JND units), whether the female was flushed from her nest during the experiment (yes or no), the nest age (days) at the time of the experiment, clutch size (eggs), and the laying date for each manipulated nest. Parameter estimates with confidence intervals that do not overlap zero are italicized.

species	parameter	estimate	s.e._adjusted_	LCL	UCL	*z*	importance
Blackbird	(intercept)	2.22	1.69	−1.15	5.58	1.29	—
	*blue-green to brown*	*0.46*	*0.18*	*0.11*	*0.82*	*2.55*	*1.00*
	flushing^a^	−1.14	1.03	−3.33	0.30	1.10	0.75
	chromatic contrast	0.07	0.20	−0.37	0.96	0.33	0.23
	less UV to more UV	−0.10	0.30	−1.46	0.59	0.32	0.22
	achromatic contrast	<0.01	0.03	−0.09	0.17	0.22	0.18
	nest age (days)	<−0.01	0.04	−0.26	0.23	0.05	0.12
	laying date	<−0.001	<0.01	−0.05	0.04	0.06	0.12
	green to purple	<−0.01	0.09	−0.54	0.51	0.02	0.12
	clutch size (eggs)	<0.0001	0.15	−0.85	0.85	<0.0001	0.12
Robin	(intercept)	−3.72	6.68	−16.99	9.55	0.55	—
	*blue-green to brown*	*2.07*	*0.95*	*0.17*	*3.96*	*2.14*	*1.00*
	flushing^a^	1.48	1.66	−0.53	5.44	0.88	0.60
	nest age (days)	0.12	0.21	−0.16	0.76	0.59	0.41
	less UV to more UV	−0.93	1.17	−3.87	0.66	0.79	0.58
	clutch size (eggs)	0.37	0.77	−0.85	3.09	0.48	0.33
	achromatic contrast	−0.11	0.21	−0.82	0.22	0.50	0.36
	green to purple	0.03	0.21	−0.74	1.12	0.16	0.18
	laying date	<−0.01	0.02	−0.13	0.06	0.26	0.20
	chromatic contrast	<−0.01	0.28	−1.57	1.49	0.02	0.13

^a^A positive effect estimate indicates that flushed females were more likely to reject the foreign egg.

We found that these hosts differed in their discrimination abilities, such that robins had a significantly stricter decision boundary than blackbirds (blackbird: median = 0.44, inter-quartile range = 0.28; robin: median = 2.78, inter-quartile range = 1.56; *r* = 1.00, slope difference = 1.99, CI_0.95_ = 1.98 to 1.99, *n* = 10^8^, *p* < 0.0001; figure 3).

## 4. Discussion

We provide experimental evidence that host response to parasitic eggshell colour is not solely based on the perceived colour difference between their own and parasitic eggs as previously thought. Instead, both host species were biased toward rejecting brown eggs and accepting blue-green eggs regardless of the absolute perceived difference in coloration between those foreign eggs and their own. By contrast, neither species predictably responded to artificial eggshell colours. These findings suggest that, from perception to action, host recognition is tuned to and within the confines of natural variation in avian eggshell colours (table 1). Specifically, hosts preferentially reject brown parasitic eggs. Our findings illustrate that host responses are predictable by biologically relevant stimuli, while their responses are not predictable by irrelevant, artificial, stimuli. Although a multiple threshold decision rule can explain host responses to foreign eggs displaying a range of novel eggshell colours (table 1), the single threshold decision rule we document is a much stronger explanation for hosts' responses. These findings highlight an unexplored cognitive mechanism underlying host egg recognition and illustrate that both sensory reception and cognitive processes are critical for host perception.

Despite similar responses, we found that these two hosts' responses differed in strength (figure 3*a*,*c*). These differences may be due to the greater range of natural variation in blackbird eggshell appearances (see inset black dots in, figure 2*b,c*), extrinsic environmental variables, or the blackbird's shared evolutionary history with the robin; however, we find the latter particularly unlikely because both egg appearance [42,43] and responses to parasitism [44] can change within decades. Instead, it is very likely that these differences relate to these hosts' adaptations to different types of parasitism. Foreign egg discrimination in the blackbird has evolved in response to either conspecific [18] or cuckoo [19] eggs that display a similar range of colours (electronic supplementary material, figure S2). By contrast, robins are parasitized by brown-headed cowbirds, *Molothrus ater* (hereafter cowbird) that lay eggs distinct from the robin's in size, colour, and pattern [45] (figures 2*c* and 3*c*), which may have resulted in the stricter decision boundary that we detected (figure 3*c*). Thus within a set of natural eggshell colours, discriminating a relevant threat is clearer for the robin. By contrast, eggshell colours that do not occur in nature (e.g. green to purple) are irrelevant and neither host produced predictable responses along this artificial gradient (table 1).

As with other types of discrimination [46,47], a host's egg discrimination ability should depend on various factors including sensory systems, cognitive abilities, coevolutionary history, and individual experience [4]. The patterns in the predictability of host responses to natural and artificial egg colours that we detected may suggest a role of learning in colour-based decisions. Studies such as ours, which quantify responses of wild animals to unconditioned stimuli, inevitably include responses from individuals with varied levels of experience and ability, and this provides a biologically meaningful estimate of stimulus response [48]. Although we found no evidence that within-season experience influenced host response (table 2), we acknowledge the possibility that prior experience with experimental or real brood parasitism by individuals in our study population may have affected an individual's response [10–12]; however, in the vast majority of hosts, including blackbirds from our study population [49], prior experience did not influence host responses (see references in [50]). Future research would benefit from examining the role of learning and prior experience by studying systems where both males and females reject (e.g. *Icterus galbula* or *Sturnus vulgaris*). In such systems, males and females may have different prior experience with egg colours, allowing researchers to differentiate prior experience from cognition.

Single and multiple threshold decision rules are not mutually exclusive. Rather, both represent cognitive processes in a host's arsenal within coevolutionary arms races. Thus, these findings do not contradict previous comparative projects and experiments that have found that the absolute perceived differences between host and parasitic egg colours are important predictors of host responses (e.g. [8,13,51,52]), particularly if they satisfy two conditions: the foreign eggshell colour aligns with the natural blue-green to brown gradient of colours found on birds' eggshells [16] and is predominantly located on the rejection side of a host's decision boundary. Many studies have used disparately coloured eggs to explore the limits of host perception [17,22,53,54], and our study provides a conceptual framework to understand why using artificially coloured foreign eggs can produce mixed results [53,55] (electronic supplementary material, figure S3). Future research would benefit from identifying decision boundaries by thoroughly sampling across a host's entire sensory space.

We do not necessarily expect to find such decision rules in all host species. Some hosts have been found to disruptively select cuckoo eggshell coloration [56], which suggests that these hosts do not discriminate between blue-green and brown eggs in the same way we have documented here, but instead could use a multiple threshold decision rule (e.g. reject both bluer and browner eggs). Similarly, if all hosts preferentially reject brown eggs, the blue-green cuckoo eggshell morph would most likely be more common than it actually is in nature [57]. However, similar decision rules may be a pervasive feature of host egg discrimination, potentially explaining why some studies have found that ultraviolet and short wavelength-sensitive quanta catch explain host responses while absolute perceived differences do not (figure 1 in [17]). Our findings suggest that brown coloration can serve as a supernormal stimulus for eliciting higher egg rejection rates than other colours. Accordingly, experimental findings from other hosts illustrate that these colours are rejected at high rates [17,53] while non-mimetic blue and green eggs are typically accepted [17,58], including the main 25 hosts of the common cuckoo *Cuculus canorus* [59]. Finally, more frequently parasitized hosts not only have greater conspecific variation in blue-green eggshell colour, but also generally have more intense blue-green eggshell coloration than less frequently parasitized hosts [60]. This evolutionary pattern would be expected if single threshold decision rules were more pervasive, but would be unexpected if hosts base rejection decisions on multiple thresholds.

Future research should determine the underlying mechanism behind this single threshold decision rule. One possibility is that hosts of avian brood parasites use colour categorization for egg discrimination. Colour categorization enables an organism to group stimuli along a discriminable gradient into distinct categories [61,62] and is characterized by a heightened discriminability between categories [62,63] (electronic supplementary material, figure S4). This mechanism can aid the decision-making process for unfamiliar tasks or when information is uncertain [63–65] and can increase the speed, accuracy, and certainty of choices, while reducing the requirements for neural processing [48,65]. Previous research has suggested that colour categorization could explain egg recognition by the tawny flanked prinia *Prinia subflava* [66], and that to detect colour categorization, researchers should compare behavioural responses to the predictions of visual models [67]. We provide that initial test and found a sharp decision boundary similar to other studies on categorical perception [48,68], but future studies should investigate the other criteria for categorical perception [62,69,70]. The single threshold decision rule is adaptive for the robin facing a parasite that lays browner eggs; however, it is unclear if this behaviour is adaptive for the blackbird that could encounter parasites laying eggs that are either more blue-green or browner than their own. These findings could suggest a cognitive constraint or that browner blackbird eggs are more likely parasitic.

Our findings illustrate that host responses are shaped by both the natural range of phenotypic variation and the sensory-cognitive constraints on host defences, and demonstrate that some hosts have strong rejection biases for specific colours (figure 3). We encourage further exploration of host responses across phenotypic spaces, and our experiment provides an approach for examining these relatively unexplored cognitive mechanisms that will advance our understanding of the underlying cognitive mechanisms of egg recognition and brood parasite–host coevolution more generally. Moreover, our work demonstrates that when attempting to understand recognition systems, natural variation in phenotypes should be considered. Finally, and most importantly, we illustrate the value of asking basic questions even in long-standing and well-established fields.

## Supplementary Material

Electronic supplementary material

## Data accessibility

Data are available online at http://dx.doi.org/10.5061/dryad.mm3kr [71].
