# Supplementary material for: Egg discrimination along a gradient of natural variation in eggshell coloration
Source: Proc Biol Sci. 2017 Feb 8;284(1848):20162592. doi: 10.1098/rspb.2016.2592 (PMC5310612; doi:10.1098/rspb.2016.2592)
Supplement: Electronic supplementary material [file rspb20162592supp1.pdf]

Egg discrimination along a gradient of natural variation in eggshell coloration

Daniel Hanley, Tomáš Grim, Branislav Igic, Peter Samaš, Analía V. López, Matthew D. Shawkey, and Mark E. Hauber

Proceedings of the Royal Society B

ELECTRONIC SUPPLEMENTARY MATERIAL

Contents:

Extended materials and methods

a) Further details on statistical parameters

b) Experimental egg models

c) Colour measurements and perceptual models

d) Chromaticity diagrams

e) Additional statistical details

f) Host discrimination ability

Supplementary References

figures S1-S4

tables S1-S3

Extended Materials and Methods

(a) Further details on statistical parameters

We estimated laying dates (1 = 1 January 2014) based on daily observations, clutch sizes, completion dates, or hatching dates assuming that both species lay one egg per day with a 13 day incubation period for the blackbird and a 14 day incubation period for the robin [S1–S3]. The age of each nest at the time of manipulation (hereafter, nest age) was calculated relative to the clutch completion date (day 0; mean nest age in days ± SD; blackbird = 3.04 ± 2.53 days, robin = 1.46 ± 2.67 days). Clutch sizes (blackbird: 4.43 ± 0.07 eggs, robin: 3.31 ± 0.08 eggs) were typical for the studied populations for both species [S4,S5].

All rejections in the robin were of the foreign egg models, while in two cases blackbirds made rejection errors [S6]. In these cases we found a host egg rejected instead of the artificial

egg [*sensu* S7], and these nests remained active. Although we include both rejections of the model egg and rejection errors as 'rejections' in all analyses, the exclusion of these two cases produced nearly identical and statistically consistent results (therefore these reanalyses are not shown). Hosts were considered 'acceptors' when the foreign egg and all of their own eggs remained incubated until the end of this period. We did not include nest desertion (i.e., abandonment) as a response to experimental parasitism and removed both deserted and predated nests from all analyses [S8]; therefore, here we have used the general term 'rejection' to refer to host responses where an egg disappeared from a nest after experimental introduction. Recent experimental research has shown that desertion is not a response to parasitism in this and other European populations of the blackbird [S3,S8–S10], and only a single robin pair abandoned its nest during this study; nonetheless, our rationale is the same [S9], these mid-sized hosts are able to grasp these models to remove them [see Video S1 from, S11].

(b) Experimental egg models

To assure rejection responses were possible, we used artificial plaster eggs similar to those used in previous experiments in both populations [S3,S4,S8,S12,S13]. The size of the eggs (mean ± SD: 22.5± 0.36 x 16.8 ± 0.29 mm, N = 82) used for blackbirds matched those of cuckoo *Cuculus canorus* eggs found in common redstart nests *Phoenicurus phoenicurus* (mean ± SD: 22.2 ± 1.0 x 16.8 ± 0.6 mm, N = 33) [data from, S14], and the size of the eggs used in robin nests (mean ± SD: 22.7 ± 0.83 x 17.4 ± 0.58 mm, N = 52) approximated those of brown-headed cowbirds (mean ± SD: 21.1 ± 1.1 x 16.47 ± 0.7 mm, N = 113) [data from, S15]. Importantly, these same egg models were successfully used in previous egg-rejection studies in both species and populations [S3,S4,S8,S12,S13], which ensures that rejection responses were possible and that

responses were not constrained by the dimensions, material, or mass [S11,S16]. Similarly, it was important that the experimental eggs used on both species were similarly immaculate and similarly coloured to aid interpretation and comparability of our findings.

We originally formulated the paint mixtures to approximate avian perceived eggshell colours based on freshly abandoned natural blackbird eggs found in 2013 as well as the full range of natural avian eggshell colours [S17], and we also created an additional mixture that generated colours along an gradient of variation that was orthogonal to the natural egg colour range within the avian colour space (figure 2). These abandoned eggs were not used as an estimate of host eggshell colour; they were only used to formulate paint mixtures. To create these mixtures we used combinations of Koh-i-Noor Hardtmuth A.s. (České Budějovice, Czech Republic) high-quality acrylic paints: brown light (0640), khaki (0530), permanent green (0520), red light (0300), turquoise (0460), and ultramarine (0410). Then prior to the 2014 field season, each foreign egg model was hand-painted with a unique paint mixture evenly across the entire egg surface such that its colour would have a unique position along either axis within the avian tetrahedral colour space (details below).

To determine how closely the freshly painted foreign eggs matched the natural eggshell colours or the orthogonal gradient, after the foreign eggs dried we measured each using a reflectance spectrometer and plotted its coordinates (details below) within the blackbird's tetrahedral colour space [S18,S19]. We then visually assessed if each egg corresponded to one of the continuous gradients of colour variation. If it did not, the egg was considered unsatisfactory and we repainted it until it did correspond with one of the two colour gradients. The exact coordinates for each foreign egg used in our experiment were statistically controlled for as a covariate in every analysis.

(c) Colour measurements and perceptual models

For each species, we measured the reflectance of foreign and abandoned eggshells between 300–700 nm using an Ocean Optics USB 2000 spectrometer (Ocean Optics, Dunedin, Florida), a pulsed xenon light source (PX-2) for blackbirds and a Deuterium Tungsten lamp (DT-mini) for robins, and a white reflectance standard (WS-1). The blackbird and robin colour datasets (foreign egg models and natural eggs) were measured using two separate sets of equipment to assure the comparability of our colour data (i.e., host versus foreign egg colour). During the course of our fieldwork we collected freshly abandoned blackbird (N = 54) and robin (N = 18) eggs, from 24 and 10 clutches respectively. To avoid the potential confounds of annual eggshell colour differences [S20,S21], we only used these freshly abandoned eggs for estimates of perceivable differences in coloration. The mean host eggshell colour was established by first averaging the reflectance spectra of eggs within each abandoned clutch, and then taking the mean reflectance spectra of these clutches. All natural and foreign eggs were measured at three random locations over the entire egg surface or across the equatorial region, for blackbirds and robins respectively. These raw reflectance spectra were analysed using the 'pavo' R package [S22]. All reflectance spectra were smoothed using a locally weighted polynomial with a 0.25 nm smoothing span, and averaged for each egg.

We modelled the relative sensitivities [S23–S25] of the blackbird's four photoreceptors with peak sensitivities at 373.0, 453.5, 504.3, and 557.2 nm [S26] and accounting for their oil droplet cut-offs at 330, 414, 515, and 570 nm respectively [S23]. We estimated achromatic quantum catch as the sum of the largest two cones. To quantify quantum catches for each photoreceptor [S27], we integrated the product of eggshell reflectance, blackbird spectral

sensitivities, and standard daylight illumination scaled for bright viewing conditions (10,000) across the avian visual spectrum (i.e., 300–700nm). To generate avian tetrahedral colour spaces we used relative quantum catch estimates [S19,S27]. Then, for each species, we estimated the discriminability between the average host eggshell colour and the perceived colours of each foreign egg using a neural noise-limited visual model [S25,S28]. This model incorporated the quantum catches of each photoreceptor, while correcting for an experimentally derived signal-to-noise ratio such that the Weber fraction of the long-wave-sensitive cone was 0.1 [S29], and accounting for the abundance of cones and the principal member of the double cone [S23] of the blackbird. These calculations were performed for the four cone types and for the double cone estimates, and produced estimates for chromatic and achromatic contrast [S25,S30] between the average perceived colour of a host's egg and the foreign egg models in units of just noticeable difference (hereafter JND).

(d) Chromaticity diagrams

In addition, to the perceptual differences between host and foreign eggs (i.e., the multiple threshold decision rule) we were interested in the perceptual, directional differences within their colour space. That is, an infinite number of colours could differ from host egg colours by any particular JND value (e.g. 2 JNDs), but hosts may not respond to all of these different colours in the same way (i.e., the single threshold decision rule). Avian tetrahedral colour spaces [S19,S27] are not perceptually uniform, meaning the distance between two stimuli within the colour space does not directly translate into perceptual differences. Therefore, including the coordinates of foreign eggs within the avian colour space in our analyses would contain information on their

directional differences, but the perceptual differences (e.g., Euclidean distances) between host eggs and these foreign eggs across the colour space would not be comparable.

To overcome this challenge and to account for the directionality of differences, we summarized perceivable variation in colour using perceptually uniform chromaticity diagrams [S31]. These chromaticity diagrams were calculated using the JND in colour between all experimental eggs and the mean colour for each species. These colour spaces were calculated such that, for each species, the species' average eggshell colour was set as the origin (i.e. zero on all three gradients). Within these chromaticity diagrams, the coordinates of each foreign egg represented the JND between that foreign egg and the mean host egg colour along each respective gradient. This approach allowed us to test the perceptual distances and their directionality, because along any gradient foreign eggs could have values greater (positive) or lower (negative) than the hosts' average eggs (e.g., an egg could differ by 1.9, 0.8, and −0.59 JND on the x, y, and z gradients respectively). When calculated in this way, the Euclidean distance between the origin and each point equalled that pair's JND in colour. We used the Cartesian coordinates from these chromaticity diagrams to examine both directional differences from the hosts' average eggshell colours and the perceptual differences of these comparisons. The Cartesian coordinates from these perceptually uniform chromaticity diagrams spanned non-noticeable and noticeable differences (i.e., $-\infty \leq \text{JND} \leq \infty$) along the two intentionally manipulated gradients (figure 2), but not the z gradient (ultraviolet variation) that was unintentionally manipulated by our treatment (figure S1). Importantly, all Cartesian coordinates were controlled for as covariates in our analyses, thus although ultraviolet variation did not span both negative and positive JNDs, we were able to control for the actual variation in each coordinate for the artificial eggs that were presented to each individual. In an absolute sense (i.e.,

the chromatic JND), 93% of the foreign eggs used on the blackbird and 100% those used on the robin were noticeably different (JND ≥ 1) from the hosts' average eggshell colours.

(e) Additional statistical details

Whole model significance was assessed by comparing a parameterized model with a null model including only an intercept using a test assuming asymptotic chi-squared distribution [S32]; while, for model coefficients we assessed significance using likelihood ratio tests [S32–S35]. To illustrate model fit we present Nagelkerke's R^2 and the small sample size corrected Akaike's Information Criterion (AIC_c) [S36,S37]. We examined potential interactions between chromatic JNDs and the x, y, and z Cartesian coordinates. These were non-significant and therefore not included in the global model; however, the significances, relative importance, and direction of parameter effects were the same. Similarly, we considered the possibility that laying date had a quadratic relationship with host response, but this did not influence our final models. Laying date controlled for the possibility that as the season progresses hosts become more experienced with parasitic eggs of if older experienced birds initiated nests earlier [S38]. Statistical parameters were chosen because each has the potential to impact our study species' response [S8,S11,S13,S39,S40].

We established a candidate set based on the relative likelihood of potential models. Specifically, models with evidence ratios greater than 1/8 were considered reasonable and we included these models in the candidate set [S41,S42]. Although other methods are available for establishing a candidate set, this method is recommended [S41,S42] and produced very similar results to alternative approaches (e.g., based on delta AIC_c and cut-offs and the 95% candidate set). We averaged models in this candidate set using the '*MuMIn*' package version 1.13.4 [S43].

The relative importance of each predictor of host response was calculated as the sum of AIC_c weights over all the models in the candidate set where that predictor occurs, setting the effect of a parameter at zero if it was not included in a particular model within the candidate set, to avoid biasing our model averaged estimates away from zero [S41].

(f) Host discrimination ability

To examine if blackbirds and robins expressed different discrimination abilities to experimental parasitism we compared the regression coefficients (i.e., slopes) of their predicted response curves, with respect to the blue-green to brown colour variation. To compare these parameters, we employed a non-replacement subsampling approach [S44–S46]. Specifically, we randomly selected 90% of the blackbird and robin data respectively and reran the GLM testing the single threshold decision rule, recording the regression coefficient (i.e., slope) for blue-green to brown variation repeatedly (10,000 times). This approach can consistently estimate statistic distributions under conditions where the bootstrap estimation would fail [S44]. The selection of subset size can be important for this approach [S47,S48], and we chose 90% because the subsampled distributions of blue-green to brown variation parameter estimates were stable for this value and approximated that of the entire (100%) dataset. We assessed normality using the '*ks.boot'* function in the 'Matching' package version 4.8–3.4. These findings were corroborated using bootstrap estimates [S46,S49].

Due to the computational challenges of examining these subsampled and bootstrapped estimates (n =100,000,000), we conducted these nonparametric tests using the high performance cluster provided by MetaCentrum/CERIT-SC. This is a network of computers that have been

made available by the Czech Education and Scientific Network and numerous participating universities within the Czech Republic.

Supporting References

S1. Snow D, Perrins CM, Doherty P & Cramp S 1998 *The complete birds of the western Palearctic: on CD-ROM*. Oxford: Oxford University Pres.

S2. Collar N 2005 American Robin (*Turdus migratorius*). In *Handbook of the Birds of the World Alive* (eds J. del Hoyo A. Elliott J. Sargatal D. A. Christie & E. de Juana), Barcelona: Lynx Edicions.

S3. Hanley D, Samaš P, Hauber ME & Grim T 2015 Who moved my eggs? An experimental test of the egg arrangement hypothesis for the rejection of brood parasitic eggs. *Anim. Cogn.* **18**, 299–305.

S4. Aidala Z, Croston R, Schwartz J, Tong L & Hauber ME 2015 The role of egg-nest contrast in the rejection of brood parasitic eggs. *J. Exp. Biol.* **218**, 1126–1136.

S5. Samaš P, Grim T, Hauber ME, Cassey P, Weidinger K & Evans KL 2013 Ecological predictors of reduced avian reproductive investment in the southern hemisphere. *Ecography* **36**, 809–818.

S6. Røskaft E, Moksnes A, Meilvang D & Honza M 2002 No evidence for recognition errors in Acrocephalus warblers. *J. Avian Biol.* **1**, 31–38.

S7. Marchetti K 1992 Costs to host defence and the persistence of parasitic cuckoos. *Proc. R. Soc. B* **248**, 41–45.

S8. Samas P, Hauber ME, Cassey P & Grim T 2014 Host responses to interspecific brood parasitism: a by-product of adaptations to conspecific parasitism? *Front. Zool.* **11**, 34.

S9. Soler M, Ruiz-Raya F, Roncalli G & Ibáñez-Álamo JD 2015 Nest desertion cannot be considered an egg-rejection mechanism in a medium-sized host: an experimental study with the common blackbird *Turdus merula*. *J. Avian Biol.* **46**, 369–377.

S10. Hauber ME, Samaš P, Anderson MG, Rutila J, Low J, Cassey P & Grim T 2014 Life-history theory predicts host behavioural responses to experimental brood parasitism. *Ethol. Ecol. Evol.* **26**, 349–364.

S11. Igic B et al. 2015 Using 3D printed eggs to examine the egg-rejection behaviour of wild birds. *PeerJ* **3**, e965.

S12. Croston R & Hauber ME 2014 Spectral tuning and perceptual differences do not explain the rejection of brood parasitic eggs by American robins (*Turdus migratorius*). *Behav. Ecol. Sociobiol.* **68**, 351–362.

S13. Hanley D, Samaš P, Heryán J, Hauber ME & Grim T 2015 Now you see it, now you don’t: flushing hosts prior to experimentation can predict their responses to brood parasitism. *Sci. Rep.* **5**, 9060.

S14. Grim T, Rutila J, Cassey P & Hauber ME 2009 The cost of virulence: an experimental study of egg eviction by brood parasitic chicks. *Behav. Ecol.* **20**, 1138–1146.

S15. Underwood TJ & Sealy SG 2006 Parameters of brown-headed cowbird *Molothrus ater* egg discrimination in warbling vireos *Vireo gilvus*. *J. Avian Biol.* **37**, 457–466.

S16. Ruiz-Raya F, Soler M, Sánchez-Pérez LL & Ibáñez-Álamo JD 2015 Could a factor that does not affect egg recognition influence the decision of rejection? *PLoS ONE* **10**, e0135624.

S17. Hanley D, Grim T, Cassey P & Hauber ME 2015 Not so colourful after all: eggshell pigments constrain avian eggshell colour space. *Biol. Lett.* **11**, 20150087.

S18. Goldsmith TH 1990 Optimization, constraint, and history in the evolution of eyes. *Q. Rev. Biol.* **65**, 281–322.

S19. Stoddard MC & Prum RO 2008 Evolution of avian plumage color in a tetrahedral color space: a phylogenetic analysis of new world buntings. *Am. Nat.* **171**, 755–776.

S20. Avilés JM, Stokke BG, Moksnes A, Røskaft E & Møller AP 2007 Environmental conditions influence egg color of reed warblers Acrocephalus scirpaceus and their parasite, the common cuckoo *Cuculus canorus*. *Behav. Ecol. Sociobiol.* **61**, 475–485.

S21. Hanley D & Doucet SM 2012 Does environmental contamination influence egg coloration? A long-term study in herring gulls. *J. Appl. Ecol.* **49**, 1055–1063.

S22. Maia R, Eliason CM, Bitton P, Doucet SM & Shawkey MD 2013 pavo: an R package for the analysis, visualization and organization of spectral data. *Methods Ecol. Evol.* **4**, 906–913.

S23. Hart NS & Vorobyev M 2005 Modelling oil droplet absorption spectra and spectral sensitivities of bird cone photoreceptors. *J. Comp. Physiol. A* **191**, 381–392.

S24. Govardovskii VI, Fyhrquist N, Reuter T, Kuzmin DG & Donner K 2000 In search of the visual pigment template. *Vis. Neurosci.* **17**, 509–528.

S25. Vorobyev M, Osorio D, Bennett ATD, Marshall NJ & Cuthill IC 1998 Tetrachromacy, oil droplets and bird plumage colours. *J. Comp. Physiol. A* **183**, 621–633.

S26. Hart NS, Partridge JC, Cuthill IC & Bennett ATD 2000 Visual pigments, oil droplets, ocular media and cone photoreceptor distribution in two species of passerine bird: the blue tit (Parus caeruleus L.) and the blackbird (Turdus merula L.). *J. Comp. Physiol. A* **186**, 375–387.

S27. Endler JA & Mielke PW 2005 Comparing entire colour patterns as birds see them. *Biol. J.*

Linn. Soc. **86**, 405–431.

S28. Vorobyev M & Osorio D 1998 Receptor noise as a determinant of colour thresholds. *Proc. R. Soc. London B* **265**, 351–358.

S29. Olsson P, Lind O & Kelber A 2015 Bird colour vision: behavioural thresholds reveal receptor noise. *J. Exp. Biol.* **218**, 184–193.

S30. Siddiqi A, Cronin TW, Loew ER, Vorobyev M & Summers K 2004 Interspecific and intraspecific views of color signals in the strawberry poison frog *Dendrobates pumilio*. *J. Exp. Biol.* **207**, 2471–2485.

S31. Pike TW 2012 Preserving perceptual distances in chromaticity diagrams. *Behav. Ecol.* **23**, 723–728.

S32. Zuur AF, Hilbe JM & Ieno EN 2013 *A beginner's guide to GLM and GLMM with R: a frequentist and Bayesian perspective for ecologists*. 1st edn. Newburgh, United Kingdom: Highland Statistics Ltd.

S33. Fox J 2016 Generalized linear models. In *Applied regression analysis and generalized linear models*, pp. 379–424. Los Angeles, California: Sage Publications Inc.,.

S34. Thiele J & Markussen B 2012 Potential of GLMM in modelling invasive spread. *CAB Rev.* **7**, 1–10.

S35. Bewick V, Cheek L & Ball J 2005 Statistics review 14: logistic regression. *Crit. Care* **9**, 112–118.

S36. Peng C-YJ, Lee KL & Ingersoll GM 2002 An introduction to logistic regression analysis and reporting. *J. Educ. Res.* **96**, 3–14.

S37. Nagelkerke NJD 1991 A note on a general definition of the coefficient of determination. *Biometrika* **78**, 691–692.

S38. Lotem A, Nakamura H & Zahavi A 1995 Constraints on egg discrimination and cuckoo-host co-evolution. *Anim. Behav.* **49**, 1185–1209.

S39. Samaš P, Hauber ME, Cassey P & Grim T 2011 Repeatability of foreign egg rejection: Testing the assumptions of co-evolutionary theory. *Ethology* **117**, 606–619.

S40. Croston R & Hauber ME 2015 Experimental shifts in intraclutch egg color variation do not affect egg rejection in a host of a non-egg-mimetic avian brood parasite. *PLoS ONE* **10**, e0121213.

S41. Burnham KP & Anderson DR 2002 *Model selection and multimodel inference: a practical information-theoretic approach*. 2nd edn. New York, NY: Springer.

S42. Burnham KP, Anderson DR & Huyvaert KP 2011 AIC model selection and multimodel inference in behavioral ecology: some background, observations, and comparisons. *Behav. Ecol. Sociobiol.* **65**, 23–35.

S43. Barton K 2015 MuMIn: Multi-Model Inference. R package version 1.13.4. , http://CRAN.R–project.org/package=MuMIn.

S44. Politis DN & Romano JP 1994 Large sample confidence regions based on subsamples under minimal assumptions. *Ann. Stat.* **22**, 2031–2050.

S45. Chernick MR 2008 *Bootstrap methods: a guide for practitioners and researchers*. 2nd edn. Hoboken, New Jersey: John Wiley & Sons, Inc.

S46. Horowitz JL 2001 The Bootstrap. *Handb. Econom.* **5**, 3159–3228.

S47. Nordman DJ & Lahiri SN 2004 On optimal spatial subsample size for variance estimation. *Ann. Stat.* **32**, 1981–2027.

S48. Bickel PJ & Sakov A 2008 On the choice of *m* in the *m* out of *n* bootstrap and its application to confidence bounds for extreme percentiles. *Stat. Sin.* **18**, 967–985.

S49. Fox J 2016 Bootstrapping regression models. In *Applied regression analysis and generalized linear models*, pp. 587–606. Los Angeles, California: Sage Publications Inc.,.

S50. Grim T, Samaš P, Moskát C, Kleven O, Honza M, Moksnes A, Røskaft E & Stokke BG 2011 Constraints on host choice: why do parasitic birds rarely exploit some common potential hosts? *J. Anim. Ecol.* **80**, 508–518.

S51. Davies NB 2000 *Cuckoos, cowbirds, and other cheats*. London: T. & A. D. Poyser.

S52. Stoddard MC & Stevens M 2011 Avian vision and the evolution of egg color mimicry in the common cuckoo. *Evolution* **65**, 2004–2013.

S53. Drobniak SM, Dyrcz A, Sudyka J & Cichoń M 2014 Continuous variation rather than specialization in the egg phenotypes of cuckoos (*Cuculus canorus*) parasitizing two sympatric reed warbler species. *PLoS ONE* **9**, e106650.

S54. Honza M, Polačiková L & Procházka P 2007 Ultraviolet and green parts of the colour spectrum affect egg rejection in the song thrush (*Turdus philomelos*). *Biol. J. Linn. Soc.* **92**, 269–276.

S55. Cassey P, Honza M, Grim T & Hauber ME 2008 The modelling of avian visual perception predicts behavioural rejection responses to foreign egg colours. *Biol. Lett.* **4**, 515–7.

S56. Hauber ME, Tong L, Bán M, Croston R, Grim T, Waterhouse GIN, Shawkey MD, Barron AB & Moskát C 2015 The value of artificial stimuli in behavioral research: making the case for egg rejection studies in avian brood parasitism. *Ethology* **121**, 521–528.

S57. Harnad S 1987 Psychophysical and cognitive aspects of categorical perception: a critical overview. In *Categorical perception: the groundwork of cognition* (ed S. Harnad), pp. 1–25. New York: Cambridge University Press.

S58. Treisman M, Faulkner A, Naish PL & Rosner BS 1995 Voice-onset time and tone-onset

time: the role of criterion-setting mechanisms in categorical perception. *Q. J. Exp. Psychol. A* **48**, 334–366.

S59. Rothstein SI 1982 Mechanisms of avian egg recognition: which egg parameters elicit responses by rejecter species? *Behav. Ecol. Sociobiol.* **11**, 229–239.

S60. Brooke M de L & Davies NB 1988 Egg mimicry by cuckoos *Cuculus canorus* in relation to discrimination by hosts. *Nature* **335**, 630–632.

S61. Reeve HK 1989 The evolution of conspecific acceptance thresholds. *Am. Nat.* **133**, 407.

S62. Davies NB & Brooke MD 1989 An experimental study of co-evolution between the cuckoo, Cuculus canorus, and its hosts. I. Host egg discrimination. *J. Anim. Ecol.* **58**, 207–224.

S63. Davies NB 1996 Recognition errors and probability of parasitism determine whether reed warblers should accept or reject mimetic cuckoo eggs. *Proc. R. Soc. B* **263**, 925–931.

S64. Rodríguez-Gironés MA & Lotem A 1999 How to detect a cuckoo egg: a signal-detection theory model for recognition and learning. *Am. Nat.* **153**, 633–648.

S65. Marchetti K 2000 Egg rejection in a passerine bird: size does matter. *Anim. Behav.* **59**, 877–883.

S66. Peer BD, Robinson SK & Herkert JR 2000 Egg rejection by cowbird hosts in grasslands. *Auk* **117**, 892–901.

S67. Lahti DC & Lahti AR 2002 How precise is egg discrimination in weaverbirds? *Anim. Behav.* **63**, 1135–1142.

S68. Servedio MR & Lande R 2003 Coevolution of an avian host and its parasitic cuckoo. *Evolution* **57**, 1164–1175.

S69. Langmore NE, Hunt S & Kilner RM 2003 Escalation of a coevolutionary arms race

through host rejection of brood parasitic young. *Nature* **422**, 157–160.

S70. Holen ØH & Johnstone RA 2004 The evolution of mimicry under constraints. *Am. Nat.* **164**, 598–613.

S71. Holen ØH & Johnstone RA 2006 Context-dependent discrimination and the evolution of mimicry. *Am. Nat.* **167**, 377–389.

S72. Hauber ME, Moskát C & Bán M 2006 Experimental shift in hosts' acceptance threshold of inaccurate-mimic brood parasite eggs. *Biol. Lett.* **2**, 177–180.

S73. Stokke BG, Takasu F, Moksnes A & Røskaft E 2007 The importance of clutch characteristics and learning for antiparasite adaptations in hosts of avian brood parasites. *Evolution* **61**, 2212–2228.

S74. Langmore NE, Stevens M, Maurer G & Kilner RM 2009 Are dark cuckoo eggs cryptic in host nests? *Anim. Behav.* **78**, 461–468.

S75. Spottiswoode CN & Stevens M 2010 Visual modeling shows that avian host parents use multiple visual cues in rejecting parasitic eggs. *Proc. Natl. Acad. Sci. U. S. A.* **107**, 8672–8676.

S76. Soler JJ, Avilés JM, Møller AP & Moreno J 2012 Attractive blue-green egg coloration and cuckoo-host coevolution. *Biol. J. Linn. Soc.* **106**, 154–168.

S77. Soler M, Martín-Vivaldi M & Fernández-Morante J 2012 Conditional response by hosts to parasitic eggs: the extreme case of the rufous-tailed scrub robin. *Anim. Behav.* **84**, 421–426.

S78. Stevens M, Troscianko J & Spottiswoode CN 2013 Repeated targeting of the same hosts by a brood parasite compromises host egg rejection. *Nat. Commun.* **4**, 2475.

S79. Bán M, Moskát C, Barta Z & Hauber ME 2013 Simultaneous viewing of own and

parasitic eggs is not required for egg rejection by a cuckoo host. *Behav. Ecol.* **24**, 1014–1021.

S80. Moskát C, Zölei A, Bán M, Elek Z, Tong L, Geltsch N & Hauber ME 2014 How to spot a stranger's egg? A mimicry-specific discordancy effect in the recognition of parasitic eggs. *Ethology* **120**, 616–626.

S81. Liang W, Yang C & Takasu F 2016 Modeling the cuckoo's brood parasitic behavior in the presence of egg polymorphism. *J. Ethol.*

S82. Knoblauch K & Maloney LT 2012 *Modeling psychophysical data in R*. New York: Springer.

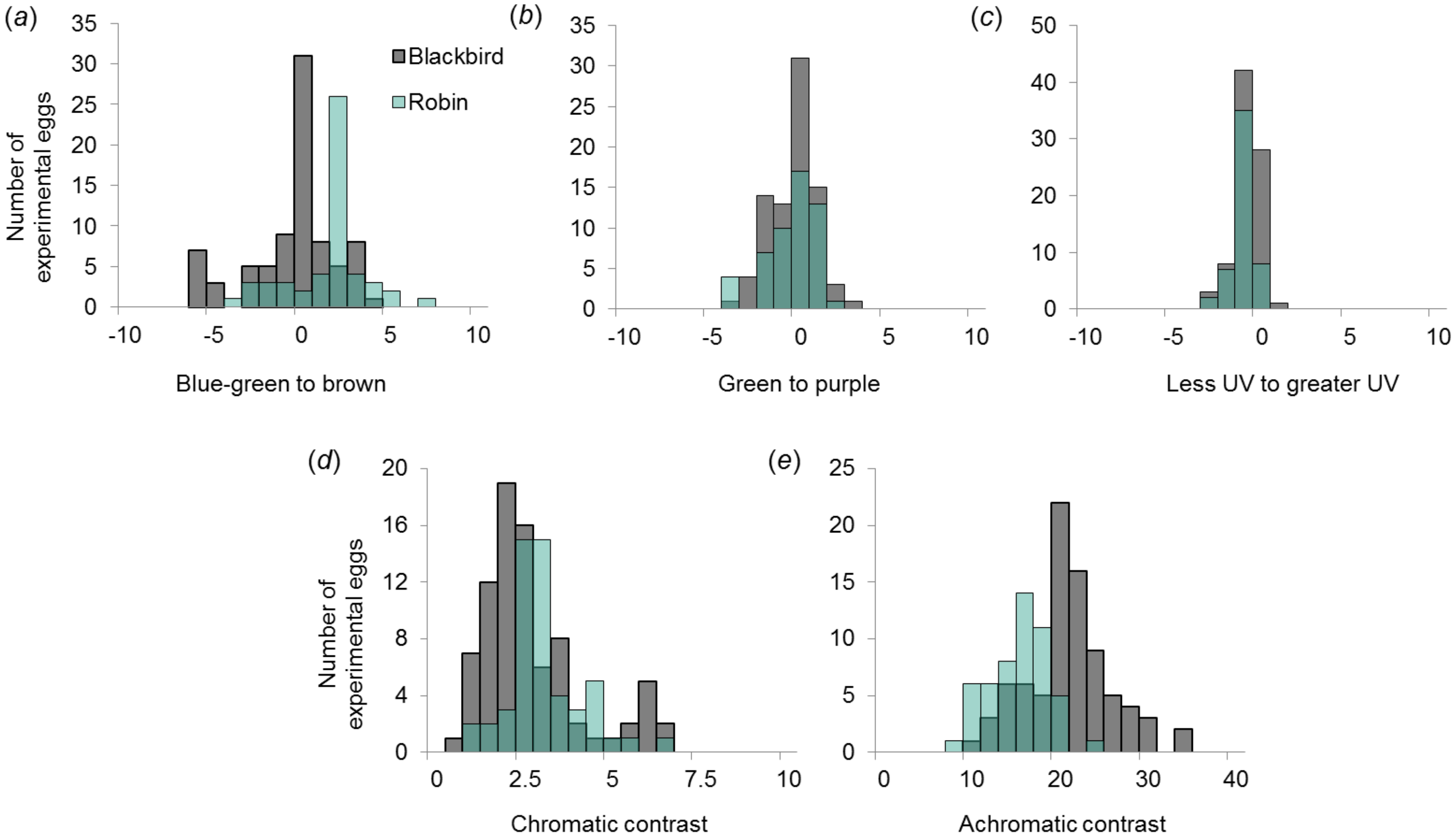

Figure S1. Our foreign eggs varied along (*a*) blue-green to brown (natural egg colours), (*b*) green to purple (artificial colours), and (*c*) less UV to greater UV gradients with respect to blackbird (grey bars) and robin (teal bars) host eggshell colours. These foreign egg

colours also spanned a wide range of absolute differences to host eggs in terms of (*d*) chromatic and (*e*) achromatic contrast (JND units). All differences are measured in just noticeable differences [S25,S30] from the hosts' own egg.

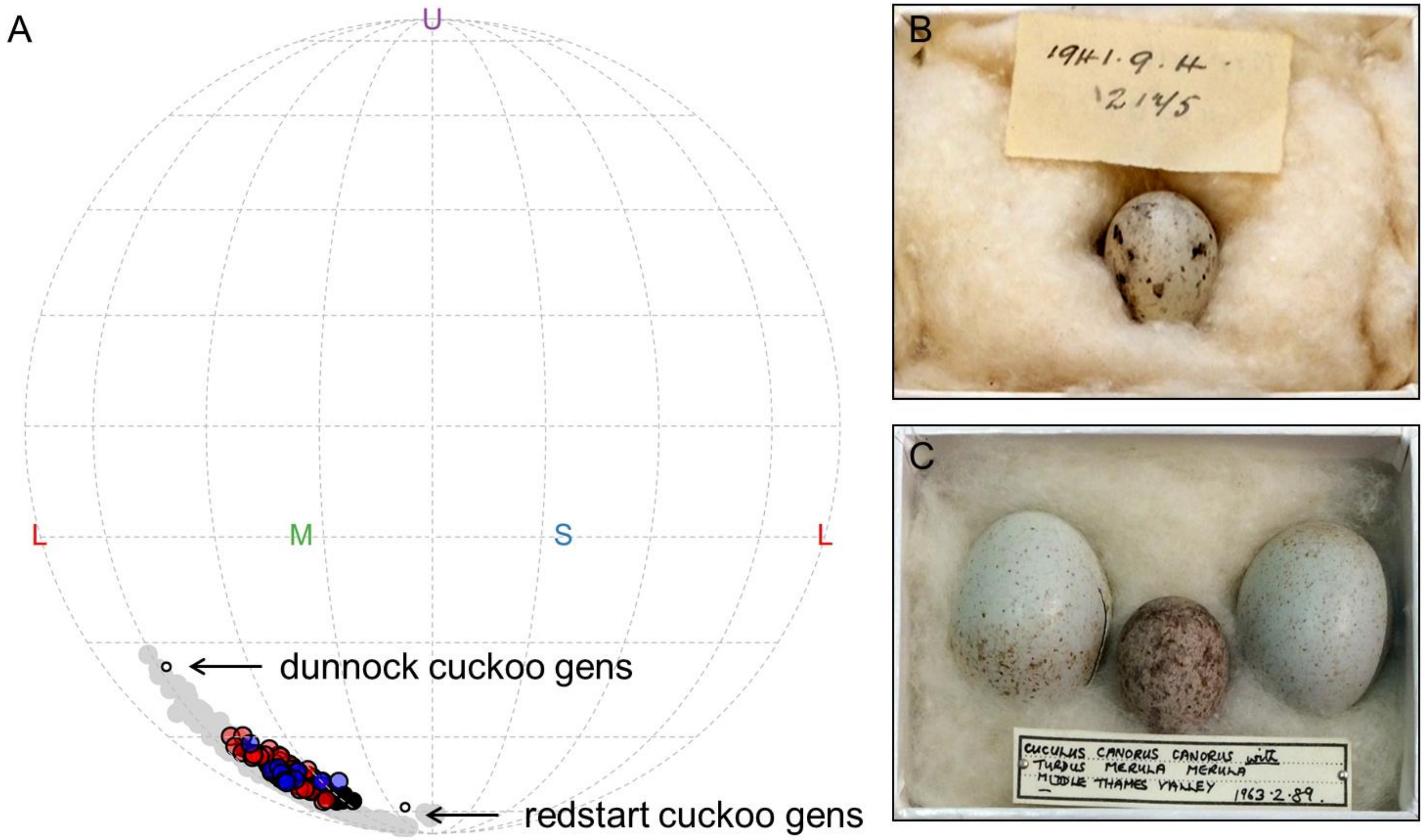

Figure S2. Blackbird egg discrimination most likely evolved in response to conspecific parasitism [S8,S50], but effects of parasitism by an extinct cuckoo gens cannot be excluded [S51]. Both parasitism forms represent similar recognition challenges because (*a*)

blackbird eggshell coloration (black dots) aligns with that of all birds' [S17] (grey dots), including: the most extreme gentes (dunnock *Prunella modularis* and common redstart *Phoenicurus phoenicurus*, white dots [S52], and gentes [S53] parasitizing reed warblers *Acrocephalus scirpaceus* (red dots) and great reed warblers *A. arundinaceus* (blue dots). These gentes parasitized blackbirds in (*b*) Oslavany, Czech Republic and (*c*) Middle Thames Valley, UK. Photography © The Trustees of the Natural History Museum, London.

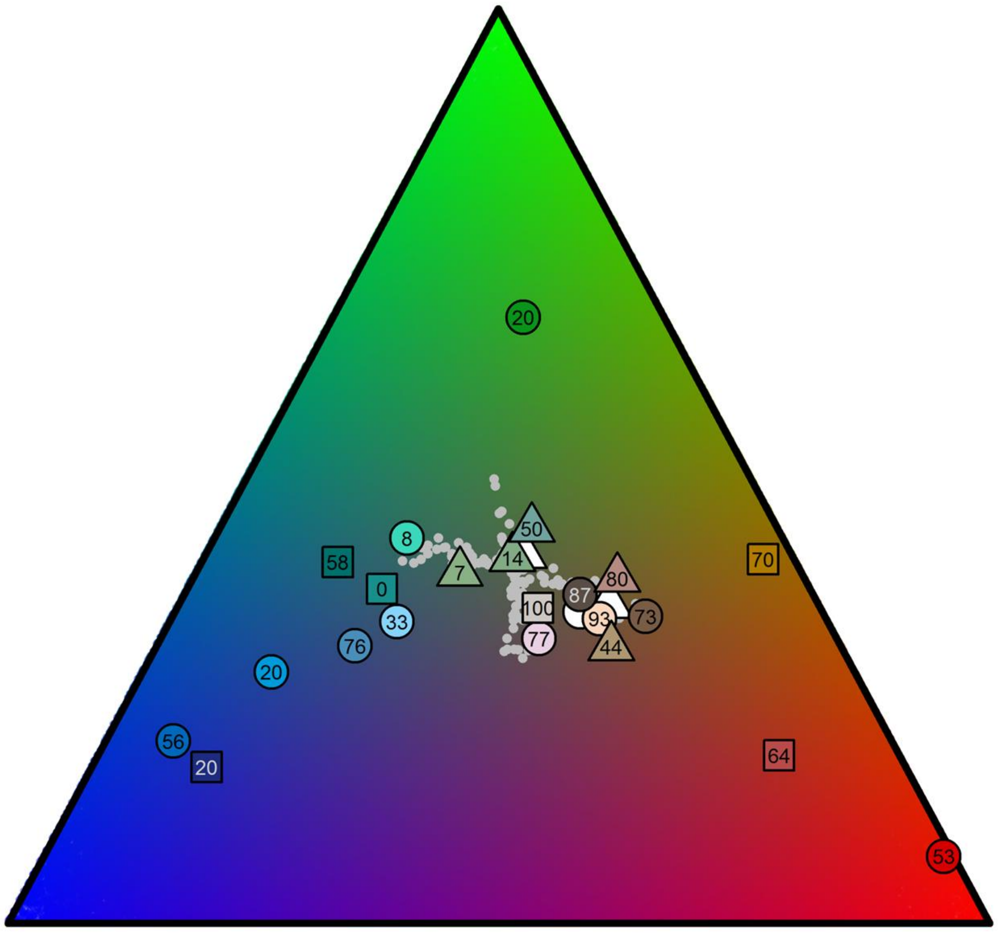

20
8
50
14
7
58
0
70
80
87
100
93
73
33
76
44
77
20
56
20
64
53

Figure S3. Here we show the experimental treatments used on robins in this study (grey dots) within the avian tetrahedral colour space (shown from above), illustrating short 'S', medium 'M', long 'L', and ultraviolet 'U' wavelength-sensitive photoreceptor stimulation. We also plot previously published experimental data on robin (squares) [S12], song thrush (circles) [S54,S55], and great reed warbler (triangles) [S56] responses to disparately coloured egg models that explored the limits of these hosts' perceptual spaces. Host rejection rates (%) are indicated inside each data point. Similar to our results (figure 3), these findings show relatively low responses (0–8%) near blue-green eggshell colours and very high responses (73–100%) on the brown side of the spectrum. Three data points representing egg models used on song thrush (87%) and great reed warblers (50% and 80%) were shifted slightly so that the rejection rates would be visible (original locations shown as a white circle or white triangles). Further research needs to carefully explore host responses outside the range we tested; however, similar to our findings these studies suggest that responses to other artificial colours are not predictable (20–76%).

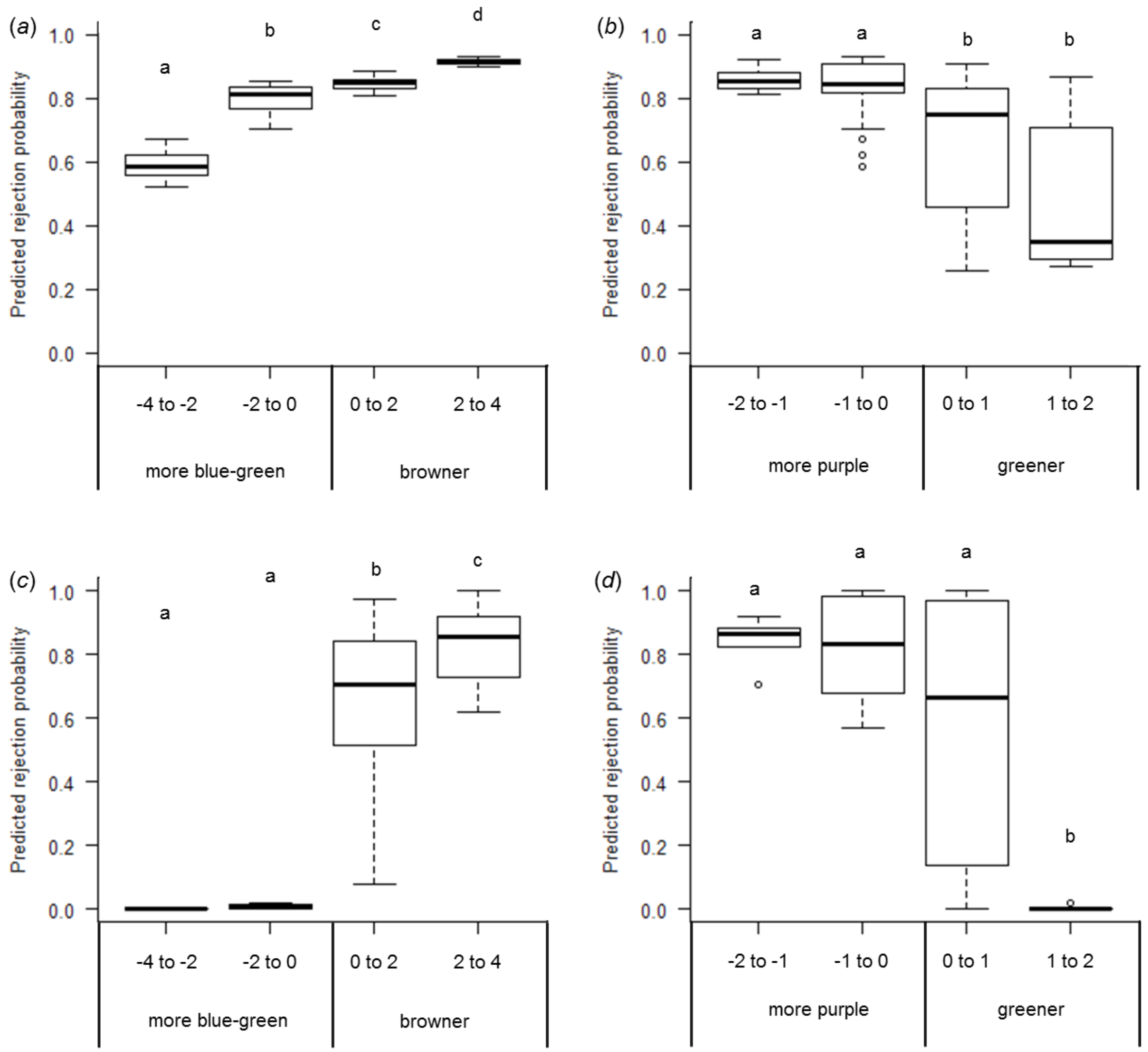

Figure S4. A defining characteristic of categorical perception is a greater ability to differentiate differences between groups than within groups, despite the absolute magnitude of those differences [S57,S58]. Here we illustrate the predicted rejection probability of eggs that (*a*, *c*) are either browner or more blue-green than (*a*) blackbird or (*c*) robin eggs and (*b*, *d*) either more purple or greener than (*b*) blackbird or (*d*) robin eggs. We depict these patterns on either side of a host's own phenotype (i.e., category), varying by level of chromatic contrast (JND) within each category. Letters above bars refer to Tukey's honestly significant difference tests.

Table S1. The approach employed by many studies makes assessing whether hosts base rejection decisions on either absolute perceived differences (e.g., multiple thresholds) or directional differences (e.g., single threshold) impossible. Studies often employ one of two common approaches: they examine directionless metrics of phenotype dissimilarity (e.g. just noticeable differences, JNDs), or they assess phenotypic variation on only one side of a host's phenotypic range. Here we provide a non-comprehensive account of how host responses have been investigated. These works are not limited to colour-based host responses because these cognitive mechanisms also apply to other traits. We indicate study focus (empirical: the focal host(s); theoretical: mathematical modelling), the phenotypic range considered (directionless: directionless metrics like JNDs or the statistical null hypothesis that disparate egg features elicit similar responses; unidirectional: one side of the hosts' phenotypic range was considered; bidirectional: both sides of the hosts' phenotypic range were considered), the phenotypic parameter used in the study (e.g., size, pigmentation, or mimicry – we distinguish theoretical estimates of mimicry from empirical estimates based on JNDs), whether this approach has the ability to detect responses based on either multiple or single thresholds, the basis for assumptions made (quote or equations, if any), and the reference. Few studies, including many of our own, were designed such that detecting directional differences was possible. For further information please see the main text. The cases are listed in chronological order.

**Host(s)**	**Phenotypic range**	**Metric used**	**Multiple thresholds or single threshold?**	**Basis of assumption**	**Reference**
Empirical: *Turdus migratorius, Dumetella carolinensis*	Directionless	Egg type[a]	multiple	"The models used give little information on the importance of degrees of difference in egg parameters…" p. 230	[S59]
Empirical: *Anthus pratensis, Acrocephalus scirpaceus, Prunella modularis, Erithacus rubecula, Motacilla alba*	Directionless	Egg type	multiple	-	[S60]
Theoretical	Directionless	Cue-dissimilarity	multiple	-	[S61]
Empirical*: Acrocephalus scirpaceus, Prunella modularis, Erithacus rubecula, Acrocephalus schoenobaenus, Troglodytes troglodytes, Emberiza schoeniculus, Fringilla coelebs, Turdus merula, Turdus philomelos, Acanthis cannabina, Carduelis chloris,Pyrrhula pyrrhula, Hirundo rustica, Muscicapa striata, Parus major, Sturnus vulgaris, Cyanistes caeruleus, Anthus pratensis, Motacilla alba, Phoenicurus phoenicurus, Ficedula hypoleuca, Oenanthe oenanthe*	Directionless	Egg type	multiple	"rejected model eggs unlike their own" p. 210	[S62]

Host(s)	Phenotypic range	Metric used	Multiple thresholds or single threshold?	Basis of assumption	Reference
Empirical*: Phylloscopus inornatus*	Unidirectional	Egg size estimate	multiple	“determine whether size differences were important in egg rejection.” p. 42	[S7]
Empirical & Theoretical*: Acrocephalus scirpaceus*	Directionless	Mimicry	multiple	“Future work needs to quantify rejection costs for different degrees of mimicry as assessed by the birds themselves and to test different host populations on either side of the predicted threshold p values for rejection” p. 929	[S63]
Theoretical	Unidirectional	Pigmentation	multiple	“further assume that the average pigmentation of cuckoo eggs, c, is greater than or equal to 0.” p. 636	[S64]
Empirical: *Phylloscopus humei*	Bidirectional	Size	single	-	[S65]
Empirical: *Sturnella magna, S. neglecta, Spizella pusilla, Pooecetes gramineus, Chondestes grammacus, Ammodramus savannarum, Spiza americana*	Directionless	Egg type	multiple	“We predicted that nonmimetic eggs should be rejected at higher frequencies than mimetic egg” p. 893	[S66]
Empirical: *Ploceus cucullatus*	Directionless	Colour atlas	multiple	"difference between two eggs’ colours was considered as the sum of the differences in lightness and chromaticity" p. 1138	[S67]

Host(s)	Phenotypic range	Metric used	Multiple thresholds or single threshold?	Basis of assumption	Reference
Theoretical	Unidirectional	Size	multiple	"We assume that cuckoo eggs are larger … than host eggs on average (or differ in a consistent direction in shape, color, or pattern)." p. 1166	[S68]
Empirical: *Malurus cyaneus*	Directionless	Egg type	multiple	"Fairy-wrens did not reject odd eggs on the basis of colour or pattern [... but deserted] clutches containing an egg larger than their own" p. 158	[S69]
Theoretical	Unidirectional	Mimicry	multiple	"Mimic trait values (denoted by m) are by convention positive" p. 600	[S70]
Theoretical	Unidirectional	Mimicry	multiple	"The mimic population is monomorphic with trait value [mimicry], by convention positive" p. 378	[S71]
Empirical: *Acrocephalus arundinaceus*	Unidirectional	Trait-dissimilarity	multiple	-	[S72]
Empirical: *Vireo gilvus*	Directionlesss	Egg type[a]	multiple	-	[S15]
Theoretical	Directionless	Mimicry	multiple	"The difference in mean population egg appearance between host and parasite (|mp−mH|) can be regarded as egg mimicry"[b] p. 2214	[S73]

Host(s)	Phenotypic range	Metric used	Multiple thresholds or single threshold?	Basis of assumption	Reference
Empirical: *Turdus philomelos*	Directionless	Egg type	multiple	"all the available studies published to date have considered the degree of similarity between the coloration of brood parasitic eggs as evidence for mimicry without considering the different sensitivity towards different colours of the particular hosts. This aspect is crucial" p. 270	[S54]
Empirical: *Turdus philomelos*	Directionless	Egg type + JND	multiple	"the average discriminability […] was not associated with the rejection responses among wild song thrushes" p. 516	[S55]
Empirical: *Gerygone magnirostris*	Directionless	JND	multiple	"$D = (\Delta S_a - \Delta S_b)/ \Delta S_b$" p. 464	[S74]
Empirical: *Prinia subflava*	Directionless	JND	multiple	“we calculated discrimination values [… (jnds), reflecting the perceived degree of difference through a bird’s eyes...]” p. 8673	[S75]

Host(s)	Phenotypic range	Metric used	Multiple thresholds or single threshold?	Basis of assumption	Reference
Empirical: Acrocephalus arundinaceus, A. scirpaceus, A. schoenobaenus, Anthus pratensis, Erithacus rubecula, Fringilla montifringilla, Lanius collurio, Motacilla alba, Prunella modularis, Phoenicurus phoenicurus, Sylvia borin	Directionless	JND	multiple	-	[S52]
Empirical: *Turdus merula, T. philomelos, T. iliacus, T. pilaris*	Directionless	Egg type	multiple	-	[S50]
Empirical: *Turdus merula, T. philomelos*	Directionless	Egg type	multiple	"We are aware of the problem that terms 'mimetic' vs. 'non-mimetic' are confusing and being used inconsistently" p. 609	[S39]

Host(s)	Phenotypic range	Metric used	Multiple thresholds or single threshold?	Basis of assumption	Reference
Empirical: *Acrocephalus palustris, A. schoenobaenus, A. scirpaceus, Anthus pratensis, Carduelis cannabina, Chloris chloris, Emberiza citrinella, E. schoeniclus, Erithacus rubecula, Ficedula hypoleuca, Fringilla coelebs, Lanius collurio, Motacilla alba, M. flava, Muscicapa striata, Oenanthe oenanthe, Phoenicurus phoenicurus, Phylloscopus collybita, P. trochilus, Prunella modularis, Sylvia atricapilla, S. borin, S. communis, Troglodytes troglodytes, Turdus philomelos*	Bidirectional	PC scores	single	-	[S76]
Empirical: *Cercotrichas galactotes*	Directionless	Egg type	multiple	-	[S77]
Empirical: *Prinia subflava*	Directionless	JND[a]	multiple	"We then used colour and pattern analyses to calculate discrepancies in each aspect of egg appearance" p. 2	[S78]
Empirical: *Turdus migratorius*	Directionless	Egg type + JND	multiple		[S12]
Empirical: *Acrocephalus arundinaceus*	Directionless	Egg type	multiple	-	[S79]

**Host(s)**	**Phenotypic range**	**Metric used**	**Multiple thresholds or single threshold?**	**Basis of assumption**	**Reference**
Empirical: *Turdus merula, T. philomelos*	Directionless	Egg type	multiple	"rejection rates of non-mimetic cuckoo-type eggs than conspecific-like model or real conspecific eggs" p. 3	[S8]
Empirical: *Turdus merula, T. philomelos*	Unidirectional	Spot coverage (20 - complete)	multiple	-	[S10]
Empirical: *Acrocephalus arundinaceus*	Directionless	Egg type	multiple	-	[S80]
Empirical: *Turdus migratorius*	Directionless	JND	multiple	"artificially increasing the visual contrasts (...JNDs ...) between experimental [ ] eggs and the nest background would [increase] rejection rates" p. 1127	[S4]
Empirical: *Turdus merula*	Directionless	Egg type[a]	multiple	-	[S9]
Empirical: *Turdus migratorius*	Directionless	Egg type	multiple	-	[S40]
Empirical: *Turdus migratorius*	Directionless	Egg type	multiple	-	[S11]
Empirical: *Turdus merula*	Bidirectional	Egg mass	single	-	[S16]

[a] This study also manipulated or examined other aspects of eggshell appearance.

[b] The notation |mp−mH| denotes absolute value. This assumption (or similar assumptions) apply to even the most recent mathematical models considering host-brood parasite coevolution [S81].

Table S2. Generalized linear models predicting the rejection probability of foreign eggs by blackbirds and robins. Here the data were fit to a signal detection theory model based on the Gaussian cumulative distribution by specifying a probit link function [S82]. Parameter estimates and model specification is otherwise identical to table 2 (main text). We present statistical tests associated with the multiple threshold and single threshold decision rule scenario, including Nagelkerke's R^2, AICc, and AICc weight (w_i) as whole model statistics. For each parameter we show the estimate, its standard errors (SE), 95% lower and upper confidence limits (LCL and UCL), z-score, and variance inflation factor (VIF). Significant models and effects are bolded.

Host	Scenario	Parameter	Estimate	SE	LCL	UCL	z	χ2	df	*P*	VIF
blackbird	**Multiple threshold (χ^2= 6.73, R^2 = 0.12, AICc = 90.69, w_i = 0.16, *n* = 82, *P* = 0.03)**										
		Intercept	0.61	0.88	−1.10	2.29	0.69	−	1	0.56	−
		**Chromatic contrast**	**−0.21**	**0.11**	**−0.43**	**< −0.001**	**−1.94**	**3.82**	**1**	**0.05**	**1.10**
		Achromatic contrast	0.03	0.04	−0.03	0.10	0.94	0.96	1	0.33	1.10
	**Single threshold (χ^2= 14.57, R^2 = 0.24, AICc = 87.33, w_i = 0.84, *n* = 82, *P* < 0.01)**										
		Intercept	0.63	0.84	−0.98	2.28	0.75	−	1	0.45	−
		**Blue-green to brown**	**0.24**	**0.09**	**0.08**	**0.42**	**2.74**	**8.40**	**1**	**< 0.01**	**1.80**
		Green to purple	−0.04	0.15	−0.34	0.25	−0.29	0.08	1	0.77	1.52
		Less UV to more UV	−0.19	0.31	−0.78	0.39	−0.62	0.41	1	0.52	1.58

	Achromatic contrast	0.01	0.04	−0.07	0.08	0.15	0.03	1	0.87	1.36
robin	Multiple threshold (χ^2= 5.83, R^2 = 0.15, AICc = 68.94, w_i < 0.0001, n = 52, P = 0.05)									
	Intercept	−1.07	1.14	−3.39	1.15	−0.93	−	1	0.35	−
	**Chromatic contrast**	**0.49**	**0.22**	**0.08**	**0.96**	**2.20**	**5.78**	**1**	**0.02**	**1.00**
	Achromatic contrast	< −0.01	0.06	−0.12	0.11	−0.05	< 0.01	1	0.96	1.00
	**Single threshold (χ^2= 35.29, R^2 = 0.67, AICc = 44.29, w_i = 1.00, n = 52, P < 0.0001)**									
	Intercept	0.07	1.85	−4.27	3.76	0.04	−	1	0.97	−
	**Blue-green to brown**	**1.39**	**0.55**	**0.59**	**2.89**	**2.53**	**28.04**	**1**	**< 0.0001**	**1.56**
	Green to purple	−0.03	0.21	−0.43	0.40	−0.16	0.02	1	0.88	1.38
	Less UV to more UV	−0.97	0.58	−2.23	0.06	−1.68	3.36	1	0.07	1.77
	Achromatic contrast	−0.20	0.12	−0.48	0.02	−1.61	3.03	1	0.08	1.39

Table S3. Generalized linear models predicting the rejection probability of foreign eggs by blackbirds and robins. Here the data were fit to a signal detection theory model based on the Weibull cumulative distribution by specifying the complementary log-log function link function [S82]. Parameter estimates and table formatting are otherwise identical to table S2.

**Host**	**Scenario**	**Parameter**	**Estimate**	**SE**	**LCL**	**UCL**	**z**	**χ2**	**df**	***P***	**VIF**
blackbird	**Multiple threshold (χ^2= 6.43, R^2 = 0.11, AICc = 90.99, w_i = 0.07, *n* = 82, *P* = 0.04)**										
		Intercept	0.36	0.79	−1.16	1.79	0.45	−	1	0.65	−
		Chromatic contrast	−0.20	0.11	−0.43	< 0.01	−1.84	3.74	1	0.05	1.06
		Achromatic contrast	0.03	0.03	−0.03	0.08	0.84	0.82	1	0.36	1.06
	**Single threshold (χ^2= 16.13, R^2 = 0.27, AICc = 85.76, w_i = 0.93, *n* = 82, *P* < 0.01)**										
		Intercept	0.32	0.74	−1.07	1.73	0.43	−	1	0.67	−
		**Blue-green to brown**	**0.29**	**0.10**	**0.11**	**0.51**	**3.05**	**10.77**	**1**	**< 0.01**	**1.61**
		Green to purple	−0.05	0.13	−0.30	0.20	−0.37	0.14	1	0.71	1.34
		Less UV to more UV	−0.27	0.27	−0.78	0.24	−0.97	1.07	1	0.30	1.76
		Achromatic contrast	< 0.001	0.03	−0.06	0.06	0.02	< 0.001	1	0.99	1.23
robin	Multiple threshold (χ^2= 5.36, R^2 = 0.13, AICc = 69.41, w_i < 0.0001, *n* = 52, *P* = 0.05)										
		Intercept	−1.19	1.19	−3.65	0.93	−1.00	−	1	0.32	−

**Chromatic contrast**	**0.43**	**0.21**	**0.06**	**0.84**	**2.08**	**5.31**	**1**	**0.02**	**1.01**
Achromatic contrast	−0.01	0.06	−0.13	0.12	−0.12	0.01	1	0.91	1.01
**Single threshold (χ^2= 34.42, R^2 = 0.66, AICc = 45.16, w_i = 1.00, *n* = 52, *P* < 0.0001)**									
Intercept	−0.75	1.86	−5.51	3.02	−0.40	−	1	0.69	−
**Blue-green to brown**	**1.43**	**0.58**	**0.62**	**3.02**	**2.49**	**28.34**	**1**	**< 0.0001**	**1.59**
Green to purple	0.01	0.20	−0.38	0.47	0.05	< 0.01	1	0.97	1.43
Less UV to more UV	−0.92	0.52	−2.04	0.05	−1.77	3.46	1	0.06	1.79
Achromatic contrast	−0.17	0.11	−0.43	0.03	−1.60	2.67	1	0.10	1.21
